# Influence of Sleep and Sleep Deprivation on Ictal and Interictal Epileptiform Activity

**DOI:** 10.1155/2013/492524

**Published:** 2013-06-12

**Authors:** Antonio Díaz-Negrillo

**Affiliations:** Clinical Neurophysiology Unit, Infanta Elena Hospital, 28340 Valdemoro, Madrid, Spain

## Abstract

Sleep is probably one of the most important physiological factors implicated both in epileptic seizures and interictal epileptiform discharges. The neurophysiology concerning the relationship between sleep and epilepsy is well described in the literature; however, the pathological events that culminate in the seizures are poorly explored. The present paper intends to make a rigorous approach to the main mechanisms involved in this reciprocal relation. Knowledge of sleep and sleep deprivation effects in epilepsy stands as crucial in the understanding of how seizures are produced, their possible lines of treatment, and future research.

## 1. Introduction

There is a very important interaction between epilepsy and sleep. This connection is not new; since antiquity, it has been recognized that some seizures only appear during sleep, as a result of which they acquired magical significance. Hippocrates stated that a person affected with epilepsy should *“spend the day awake and the night asleep. If this habit be disturbed, it is not so good … worse of all when he sleeps neither night nor day”* [1]. 

Sleep and sleep deprivation have an influence in the onset, frequency, and semiology of seizures, as well as in EEG findings. Some seizure types have different circadian distributions, and understanding these patterns may provide useful diagnostic clues [2]. In 1885, Gowers observed in 850 institutionalized patients that 21% of seizures occurred exclusively at night, 42% only during day, and 37% interchangeably during day or night. In those terms, he classified seizures occurrence as diurnal, nocturnal and diffuse [3]. In 1890 Féré studied the times when the seizures occurred over 3 months in epileptic hospitalized patients, finding that two thirds all of seizures occurred between 8 p.m. and 8 p.m. [4]. Some years later, Langdon-Down and Russell Brain in 1929 analysed 2524 seizures in 66 patients over 6 months: 24% of seizures were nocturnal, 43% daily and 33% occurred randomly [5]. Janz was the first to describe what he called “awakening epilepsy.” In 1969, he published five articles about the chronobiology of tonic-clonic generalized seizures in 2825 patients: 44% had seizures during sleep and 33% during waking [6]. Gibberd and Bateson studied 645 patients with epilepsy, founding that sleep-related epilepsy has an ultradian pattern differentiated at the beginning and end of sleep, while seizures in epilepsy daytime waking occur preferentially in the midafternoon [7].

The interaction between sleep and epilepsy is reciprocal; sleep affects the presentation mode of epilepsy and epileptic seizures modifies the sleep pattern and contributes to its fragmentation. Sleep deprivation is not only associated with epilepsy [8]. It has been implicated in several physiopathological effects, including decreased appetite, altered memory, and learning [9, 10]. Although there is abundant evidence that sleep deprivation facilitates the onset of epileptic seizures and interictal epileptiform discharges, there are also studies that propose a critical view of this claim arguing that sleep deprivation rarely occurs isolated but is in fact frequently associated with stress, alcohol, and so forth [11, 12]. 

The discovery and use of the electroencephalograph revolutionized the understanding of the relationship between epilepsy and sleep. Interictal epileptiform discharges are more common in NREM sleep [13]. The frequency of epileptiform discharges during sleep not only increases, but also affects their morphology and distribution [14]. There are several partial and generalized epileptic syndromes associated with sleep. Recognizing them is essential in order to make an accurate diagnosis and suggest the adequate treatment.

## 2. Physiopathological Fundamentals

There is no clear explanation of the relationship between some types of epilepsy and its presentation during drowsiness, sleep, or awakening. Both sleep and some generalized seizure types share common neuroanatomical elements comprising the ascending reticular activating system, limbic system, and specific thalamic nuclei. *In vitro* electrophysiologic studies of human brain slices have reported that spontaneous activity and responses to synaptic activation in neocortex and hippocampal neurons differ in normal and epileptic tissues [15]. 

 It is well accepted that both partial and generalized interictal epileptiform discharges increase during NREM (Non Rapid Eye Movement) sleep, whereas REM (Rapid Eye Movement) sleep suppresses generalized epileptiform discharges and shows variable effects on focal discharges. Sato and Nakashima reported that hippocampus-kindled cats showed a lower electroconvulsive seizure threshold in slow-wave sleep compared with wakefulness and REM sleep [16]. The synchronous oscillations of neurons generating cortical sleep spindles, K-complexes, and slow waves during NREM sleep promote the propagation of epileptic discharges. These could be some of the most important mechanisms involved in the trigger of seizures during NREM sleep. Therefore, epileptic discharges could appear instead of sleep spindles in a cortical response to intrinsic thalamocortical inputs [17]. In fact, the relation between sleep spindles production and epileptic activity has been documented. Frontocentral areas are the most important side of spindles production and, because of the rich interconnectivity between each region of the frontal lobe, sleep may facilitate motor seizures and other types of frontal seizures. 

 On the other hand, REM sleep is defined by the EEG desynchronization and loss of muscle tone. The EEG desynchronization prevents the spread of seizures during REM sleep as in wakefulness, and the lack of muscle tone blocks clinical expression of seizures. During REM in humans and other mammalians', sleep there exists an increasing GABAergic activity which is protective of seizures [18]. Therefore, seizures during this stage occur infrequently. The protective role of REM sleep is probably mediated by GABA hypothesis and other factors, such as the desynchronization pattern and reduced muscular tonus during this sleep phase. EEG recordings generally show absence or decreased epileptiform activity in most patients. 

 Biochemical changes responsible for cerebral hypersynchrony present during certain stages of sleep may encourage propagation of discharges. As mentioned above, several mechanisms have been implicated in this processes such as loss of inhibition on some circuits or thalamocortical facilitation and propagation of epileptiform activity occurs possibly as subgroups of seizures originating in the frontal lobe. Autosomal dominant frontal epilepsy, characterized by nocturnal seizures, appears to be associated with a mutation of the A-4 subunit of the nicotinic acetyl receptor (CHNRA-4) [19]. Therefore, the participation of the acetylcholine system may be considered. Monoaminergic and cholinergic brainstem receptors reduce firing rates producing hyperpolarization in thalamocortical relay neurons, which is maximal in NREM stages 3 and 4 sleep. During REM sleep, cortical activation occurs when cholinergic brainstem afferents increase firing rates producing depolarisation in thalamo-cortical relay neurons [20, 21]. 

Sleep deprivation is also an important trigger that increases the interictal epileptiform activity, especially in the transition from wakefulness to light sleep whose action mechanism probably increases cortical excitability [22]. Transcranial magnetic stimulation studies have shown that sleep deprivation is associated with important changes in inhibition-facilitation balance in the primary motor cortex of normal subjects. Motor threshold probably reflects axon membrane excitability, because drugs act on voltage and frequency-dependent calcium and sodium channels modulate it. These modifications might have a connection with the background factors of the “activating” effects of sleep deprivation [23]. Some laboratory studies have implicated nitric oxide, as inhibitory substance of seizure, associating the action of some anticonvulsant for mediating this [24]. However, other studies place it as proconvulsant substance; as in experimental studies with genetic absence occurs at high concentrations, their concentration is high during some seizures and sleep [25]. In the present state of knowledge, the most accepted conclusion is that nitric oxide behaves as a neuromodulator with dual (proconvulsive or anticonvulsive) action [26].

As shown, there are multiple pathophysiological mechanisms linking sleep to epilepsy. Therefore, having a deep knowledge of the features involved in sleep is very essential for a proper understanding of the characteristics of this type of seizures.

## 3. Epilepsy and Circadian Rhythms

Knowledge about the circadian rhythm implication on epilepsy is relatively limited. Gaining knowledge into this factor is very important for a better understanding of the physiopathology of epilepsy and for its diagnosis and treatment. Circadian rhythms are endogenously mediated 24-hour cycles of physiological processes, including the sleep-wake cycle, hormone production, and core body temperature. These circadian rhythms are modulated by a biological clock located in suprachiasmatic nuclei. This and other anatomical structures such as retinohypothalamic tract or pineal gland are responsible for the circadian pacemaker (process C), which allows alertness during the subjective day, sleepiness during the subjective night, and an increase in sleepiness (process S), which depends on the prior time awake [27]. There are several interesting studies that show a direct relation between epilepsy and sleep-wake rhythm both in animals and humans. Studies with rats placed in constant darkness manifested spontaneous limbic seizures occurring in a mediated circadian pattern [28]. In genetic terms, it has been shown that the loss of circadian PAR bZIP transcription factors in mice may produce epilepsy [29]. 

There are numerous examples about the association between sleep and seizures in some frontal lobe syndromes (e.g., autosomal dominant nocturnal frontal lobe epilepsy) and myoclonic seizures in juvenile myoclonic epilepsy which appear most likely after awakening in the morning. Pavlova et al. described 90 seizures in 26 patients founding an increased likelihood of seizures between 15:00 and 19:00 hours (temporal epilepsy) and another peak incidence between 19:00 and 23:00 hours (extratemporal epilepsy) [30]. In 2006, Pung and Schmitz studied the circadian rhythms of 20 patients with juvenile myoclonic epilepsy and 20 patients with temporal lobe epilepsy. They found very interesting differences in the circadian activity patterns of both groups. They observed that patients with juvenile myoclonic epilepsy seem to have a characteristic circadian rhythm similar to an extreme eveningness (staying up late, getting up later in the morning). However, patients with temporal lobe epilepsy could be defined as “morning types” [31]. Durazzo et al. (2008) reported on 669 seizures in 131 patients showing occipital seizures peaked between 16:00 and 19:00 hours, frontal and parietal lobe seizures peaked between 04:00 and 07:00 hours; mesial temporal lobe seizures showed two peaks (16:00–19:00 and 07:00–10:00) [32]. 

On the other hand, Hofstra et al. in 2009 presented 450 seizures in 33 patients explored with long-term intracranial EEG and video monitoring. Their results were temporal lobe seizures occurred preferentially between 11:00 and 17:00 hours, parietal lobe seizures between 17:00 and 23:00 hours, and frontal lobe seizures between 23:00 and 05:00 hours [33] (see Table 1). Analyzing the studies mentioned, we can see that seizures originated in the temporal, parietal, and occipital lobes tend to occur in the afternoon. Instead of those originating in the frontal lobe tend to occur during the night.

 Other parameters like body temperature, heart rate, and hormonal levels have been studied and related to seizures [34]. Several authors have reported reduced heart rate variability in people with chronic epilepsy. Likewise, normal baseline levels of cortisol have been described with postictal elevations, or elevated baseline prolactin levels have been observed and levels may rise further after seizures [35]. 

As we have seen, several studies have indicated the interaction between circadian rhythms and epilepsy. The results discussed previously suggest that some physiological factors with a circadian distribution can influence the presentation of numerous types of seizures. We believe that their identification is very important in order to help prevent seizures by modifying these factors.

## 4. Sleep and Ictal-Interictal Epileptiform Activity

The trigger capacity of NREM sleep on seizures and interictal epileptiform abnormalities has been widely tested. By contrast, REM sleep has inhibitory properties. Passouant named REM sleep “anticonvulsant sleep” because of its general inhibitory effect of anomalies, both ictally and interictally [36]. This effect is characteristic in hypsarrhythmia and also the epileptiform activity of the Lennox-Gastaut syndrome. During this sleep, phase generalized tonic-clonic seizures never occur. However, when localized anomalies observed during REM sleep exist, there is a good correlation with the topographic area.

 In 1947, E. L. Gibbs and F. A. Gibbs observed in 500 patients that while 36% had interictal epileptiform discharges during awakening, 82% did so during sleep. They checked sleep-actives-independent foci in some patients, and the probability of interictal epileptiform discharges was highest in patients with acute psychomotor seizures [37]. Few years later, Gloor et al. observed in 300 patients that 57% had more interictal epileptiform discharges during sleep [38]. In 1972, Niedermeyer and Rocca reported that around 30% of patients with refractory temporal lobe epilepsy had interictal epileptiform discharges only during sleep. In these patients EEG is characterized by an increased frequency of spikes at sleep onset, being highest during slow sleep and decreasing in REM [39]. Frontal lobe seizures begin during sleep more frequently than temporal lobe seizures. 

NREM sleep not only increases focal discharges. It also increases generalized discharges. In idiopathic generalized epilepsies, sleep increases the number of seizures and interictal epileptiform discharges. The spikes frequency increases when sleep starts and during slow sleep, decaying in REM sleep. The most common interictal abnormality in these patients are spike-wave discharges of 4 to 5 Hz; generalized short bursts of 1 to 3 seconds sleep duration, but more frequent drowsiness. In the same line, awakening is also another physiological inductor, especially epilepsy with generalized tonic-clonic seizures and myoclonic epilepsies. The same can be said of the transition states between NREM and REM sleep, which are activators in epilepsy with generalized tonic-clonic seizures, myoclonic epilepsy, and absences. Sleep increases epileptic discharges in absences in 81–90% approximately. The spike-wave discharges at 3 Hz with continuous discharges during status are fragmented during NREM sleep. In the absence status, spike discharges and polyspike-wave can be seen isolated [40, 41]. 

In conclusion, analyzing the contributions of the authors consulted, NREM sleep has a pronounced trigger effect on different types of seizures and over interictal epileptiform discharges fundamentally secondary generalization of partial seizures, especially those of temporal lobe origin. During sleep, frontal lobe seizures occur more often than temporal lobe seizures. We believe that these are important reasons to include a period of sleep in routine EEG obtaining an increase in the diagnostic value and sensibility.

## 5. Sleep Deprivation and Ictal-Interictal Epileptiform Activity

The effects of sleep deprivation are known since long ago. The actions over cognitive and psychomotor functions, decreasing the concentration and attention, are known for all of us. If deprivation persists, diplopia, dysarthria, postural tremor, and psychotic symptoms occur. The relationship between sleep deprivation and epilepsy is very close. Malow said “Study of the effects of sleep deprivation on epilepsy may create a window of opportunity that could help decipher how seizures are triggered and facilitated, potentially providing an avenue for future interventions” [42].

Substantial evidence recognizes sleep deprivation as a precipitant of seizures. Sleep deprivation is used in some types of epilepsy to facilitate seizures for the diagnosis or presurgical evaluation of epilepsy. Sleep deprivation can promote and facilitate both seizures and interictal epileptiform discharges. It can detect epileptiform abnormalities in 35% of patients with normal EEG. This effect is especially evident in primary generalized epilepsy, independent of sleep effect [43–45]. 

There are several studies to support this thesis both in animals and humans. For example, in cats, sleep deprivation facilitates penicillin-induced seizures, and in one genetic model of absence epilepsy in rats deprived of sleep for 12 hours recorded an increased number of spike-wave discharges within first 4 hours of sleep deprivation [46, 47]. In 1962, Janz reported that sleep deprivation by alcohol-precipitated tonic-clonic generalized seizures, particularly after awakening [48]. Three years later, Mattson et al. published the following study: sleep-deprived EEGs were realized after 26 to 28 hours of wakefulness in 89 patients with a history of at least one seizure and a normal routine EEG, 34 patients with convulsive epilepsy and interictal epileptiform discharges on routine EEG, and 20 patients with other neurologic disorders (nonepileptic disorders). Interictal epileptiform abnormalities were recorded in 34%, 56%, and 0% of subjects, respectively [49]. 

During the 60s and the 70s, several studies in pilots and soldiers sleep deprived for long periods showed that seizures indeed occurred in the context of sleep deprivation but frequently associated with other factors such as stress, fatigue, alcohol, and drug abuse. In 1991, R. Degen and H. E. Degen compared the effect of total sleep deprivation on the EEGs of patients with different types of epilepsies. For most seizure types, spontaneous sleep and sleep-deprived recordings produced similar activation rates. Seizures were more likely to be activated by sleep or sleep deprivation in patients with idiopathic generalized epilepsy than partial epilepsy [50]. Two years later, Rajna and Veres observed that night sleep duration was directly correlated with seizures frequency in 14 patients with temporal lobe epilepsy. They saw that the probability of a seizure was significantly higher after a night of sleep deprivation compared with normal sleep [51]. 

A deep debate exists about whether the interictal epileptiform discharges activation produced by total sleep deprivation is due to sleep itself or because total sleep deprivation makes an independent activating effect. Some studies have proposed that total sleep deprivation does not offer greater activation than sleep alone; others claim that total sleep deprivation activates interictal epileptiform discharges independent of sleep induction. Rowan et al. described a significantly greater interictal epileptiform discharges yield following total sleep deprivation compared with routine wake; interictal epileptiform discharges were registered in 28% of the patients only following total sleep deprivation, and total sleep deprivation activated a new epileptic focus in 7% of cases [52]. In 2000, Roupakiotis et al. presented a study of 721 subjects who had a second EEG (routine EEG, drug-induced sleep, or total sleep deprivation) after an inconclusive basal EEG founding a significantly greater percentage containing interictal epileptiform discharges after total sleep deprivation as compared with a second routine record (22.6% versus 9.5%) [53]. 

As a finding, in line with Foldvary-Schaefer and Grigg-Damberger, we can say that comparative studies largely confirm that total sleep deprivation activates ictal and interictal epileptiform discharges in 23–93% of patients with definite or suspected seizures [54]. In our experience, partial sleep deprivation is useful for EEG routine producing a significant increase on epileptiform findings.

## 6. Sleep-Related Epileptic Syndromes

There are certain epileptic syndromes whose EEG abnormalities occur predominantly during sleep and which are clearly influenced by it. Fundamentally, there are partial epilepsies, which may be idiopathic, symptomatic, or cryptogenic. New diagnostic methods such as functional MRI, MRI with spectroscopy, and the tractography, and the introduction of the new antiepileptic drugs (e.g., Rufinamide in Lennox-Gastaut syndrome) have enabled innovative positions in the management of these diseases. The most important sleep-related epileptic syndromes are briefly described later.

### 6.1. Benign Childhood Epilepsy with Centrotemporal Spikes (BCECTS)

This is the most common epileptic syndrome in infancy and it can be considered a sleep-related epilepsy because 75% of all seizures occur exclusively during NREM sleep, being particularly frequent in the first third of the night [55, 56]. In nocturnal seizures, the generalization of the seizure often occurs. The EEG is usually normal during wakefulness (only 10–20% of all patients have seizures during wakefulness) and often during N1 and N2 sleep it shows abundant spikes or spike-wave localized unilaterally or bilaterally on the middle temporal and central regions. During N3, this activity tends to be multifocal, even generalized and, in some cases, when atypical evolution exists, a pattern similar to epilepsy with continuous spike-wave during slow sleep (less than 85% total NREM sleep) can be observed. During REM sleep the frequency, amplitude, and persistence of abnormalities decrease [57, 58].

### 6.2. Benign Childhood Epilepsy with Occipital Paroxysms

Childhood epilepsy with occipital paroxysms, which are blocked by the eye opening in waking, is clearly another syndrome facilitated by sleep, during which, in all phases, the presence of the paroxysms clearly increases. This facilitator feature and normality sleep architecture are different characteristics of occipital epilepsies and have a predictive value of benignity [59]. 

### 6.3. Frontal Lobe Epilepsy

Frontal lobe epilepsy usually presents seizures almost exclusively during sleep [60]. It is very important to make a correct differential diagnosis with certain parasomnias. EEG findings are crucial. Seizures originated in frontal lobe have some typical characteristics: predominant motor activity during event, short duration, minimal postictal confusion, and tendency to occur several times during the night. The most accepted thesis is that during sleep there is facilitation of frontal discharges in thalamo-cortical circuits, sometimes associated with sleep spindles and generated in thalamic nuclei [61]. Autosomal dominant nocturnal frontal epilepsy is expressed by a variety of clinical manifestations ranging from abrupt awakenings or dystonic movements, to complex motor behavior. Seizures are brief and usually occur in N2 sleep stage. The sleep architecture is normal. In some cases, there are anomalies described in “alternating cyclic pattern” [62, 63].

### 6.4. West Syndrome

West syndrome is a rare epileptic syndrome in infants. The epileptic seizures which can be observed are known as infantile spasms and EEG findings show a pattern described as hypsarrhythmia. The hypsarrhythmia shows a variation during sleep with typical EEG with multifocal spikes and high-voltage waves superimposed on a disorganized and chaotic baseline activity. During NREM sleep, amplitude increases basal activity and spike-wave discharges tend to cluster in periodic complexes. In REM sleep, epileptiform activity decreases or disappears [25].

### 6.5. Landau-Kleffner Syndrome

This syndrome is characterized by seizures initially of easy control and sensory aphasia. The EEG shows epileptiform discharges which are highest in temporal or central temporal areas. During NREM sleep these discharges tend to be continuous and disappear or be fragmented during REM sleep [64]. In the Landau-Kleffner syndrome basal EEG activity can be normal during wakefulness and find continuous spike-wave discharges during sleep. It is believed that the epileptic discharges during sleep are facilitated by the spindles.

### 6.6. Lennox-Gastaut Syndrome

Lennox-Gastaut syndrome is an epileptic encephalopathy characterized by different seizure types and increasing abnormal activity during sleep. The average age of onset is between 2 and 5 years. It is characterized by difficulty to control seizures being tonic and atonic as the most frequent. In most cases, there is a variable degree of mental retardation. Approximately half of the patients have a significant perinatal history of intraventricular haemorrhage, neuronal migration disorder, Aicardi syndrome, tuberous sclerosis, or West syndrome. The EEG characteristically shows multifocal, diffused or generalized discharges of slow spike-wave or polyspike wave. During sleep polyspike activity can be prominent and basal activity can be seen as continuous epileptiform activity. Rhythmic activity of 10 to 25 Hz occurs almost exclusively during sleep [65]. However, the only seizures consistently promoted during sleep are tonic seizures. The increased activity during sleep is associated with poorer clinical prognosis.

### 6.7. Epilepsy with Continuous Spike-Wave during Slow Sleep

This type occurs in children. Often these patients have some degree of mental retardation and/or learning difficulty. EEG findings may have multifocal discharges. Typically, the EEG during NREM sleep slows epileptiform discharges with maximal amplitude in frontal areas and continuous activity of spike-wave which fills approximately 85% of the record [66].

### 6.8. Juvenile Myoclonic Epilepsy

Juvenile myoclonic epilepsy is the most common generalized epilepsy primarily in adolescents and adults. It is characterized by myoclonic jerks (in all patients) and other type of seizures (generalized tonic-clonic, absences, etc.). The seizures occur preferentially after awakening and are very sensitive to sleep deprivation, alcohol, or photostimulation. Interictal activity is short bursts of polyspikes and polyspike-wave complexes after spontaneous or induced awakenings [67, 68]. In general, sleep architecture is affected, with decreased quality and sleep fragmentation. 

### 6.9. Epilepsy with Generalized Tonic-Clonic on Awakening

In 1885, Gowers described a group of patients whose seizures appeared after waking. Epilepsy with generalized tonic-clonic on awakening has many similarities with juvenile myoclonic epilepsy as to the age of onset or precipitating seizure factors. 90% of all seizures occur within 2 hours following waking, at any time of day. Most frequently, interictal anomalies are generalized spike-wave or polyspike-wave complexes, arrhythmic, of a few seconds of duration, with frequencies of about 3 to 6 Hz. This activity is facilitated by hyperventilation, sleep deprivation, and photostimulation [69].

## 7. Conclusions

Despite the clear relationship between sleep, sleep deprivation, and epilepsy, we know little about it. The most accepted thesis suggests that the relationship is reciprocal and that overall both conditions (sleep and sleep deprivation) have an excitatory effect on certain types of epilepsy. The tendency to circadian presentation of some epileptic syndromes and the trigger effect of NREM sleep on epileptic seizures are good examples. However, some authors question these claims, particularly those related to the effect of sleep deprivation on epilepsy. Moreover, treatment of these sleep disorders can lead to improved seizure control.

Correct understanding of issues like the process of genesis and propagation of the seizures, or sleep-related epileptic syndromes is crucial in order to make a correct diagnosis and appropriate treatment.

## Figures and Tables

**Table 1:** Results of some interesting studies that relate seizures location and most frequently time of day.

Author	Year	Number of patients	Number of seizures	Focus	Peak hour
Pavlova	2004	26	90	Temporal	15–19 h
				Extratemporal	19–23 h

Durazzo	2008	131	669	Occipital	16–19 h
				Frontal and Parietal	04–07 h
				Temporal	16–19 h and 07–10 h

Hofstra	2009	33	450	Temporal	11–17 h
				Parietal	17–23 h
				Frontal	23–05 h
